# The complete genome sequence of CrRV-Ch01, a new member of the family *Rhabdoviridae* in the parasitic copepod *Caligus rogercresseyi* present on farmed Atlantic salmon (*Salmo salar*) in Chile

**DOI:** 10.1007/s00705-018-3768-z

**Published:** 2018-02-14

**Authors:** Arnfinn Lodden Økland, Renate Hvidsten Skoge, Are Nylund

**Affiliations:** Department of Biology, University of Bergen, 5020 Bergen, Norway

## Abstract

We have determined the complete genome sequence of a new rhabdovirus, tentatively named Caligus rogercresseyi rhabdovirus Ch01 (CrRV-Ch01), which was found in the parasite *Caligus rogercresseyi*, present on farmed Atlantic salmon (*Salmo salar*) in Chile. The genome encodes the five canonical rhabdovirus proteins in addition to an unknown protein, in the order N-P-M-U (unknown)-G-L. Phylogenetic analysis showed that the virus clusters with two rhabdoviruses (Lepeophtheirus salmonis rhabdovirus No9 and Lepeophtheirus salmonis rhabdovirus No127) obtained from another parasitic caligid, *Lepeophtheirus salmonis*, present on farmed Atlantic salmon on the west coast of Norway.

**Electronic supplementary material:**

The online version of this article (10.1007/s00705-018-3768-z) contains supplementary material, which is available to authorized users.

The parasitic copepod *C. rogercresseyi* (sea louse) is a serious problem for farming of Atlantic salmon in Chile [1–3]. There are no published studies of viruses infecting *C. rogercresseyi*, but sequences of possible rhabdovirus origin (N protein, G protein and RNA-dependent RNA polymerase genes) have been detected in the parasite. However, these sequences have been suggested to be possible endogenous viral elements (EVEs), i.e., viruses integrated in the germline genome of their hosts and inherited vertically [4–14], or integrated rhabdoviral elements (IREs), i.e., “fossil” traces of extinct rhabdoviruses, and not exogenous rhabdoviruses [8]. This was also suggested to be the case for rhabdovirus-like sequences found in genomes of *L. salmonis* [8], but Økland et al. [15] showed that these EVEs were actual pieces from two different rhabdoviruses infecting this parasite. Hence, the focus of this study was to identify an exogenous rhabdovirus in *C. rogercresseyi* using EVEs/IREs available in the GenBank database (NCBI) as a starting point.

Sequences resembling partial rhabdovirus genomes were identified by BLAST search [16] using several rhabdoviruses as query sequence and limiting the search to *C. rogercresseyi* (taxid: 217165). Additional sequences where identified using BioEdit [17] to perform a local BLAST search in a database constructed from a *C. rogercresseyi* transcriptome shotgun assembly (PRJNA234316). A rhabdovirus genome sequence (11,529 nucleotides) based on three sequences (GenBank accession numbers BT075815, GAZX01041482 and GAZX01041484) was assembled using Contig Express from the Vector NTI® Suite 9.0.0 (Thermo Fisher).

The complete genome sequence (11,599 nucleotides) of the new rhabdovirus, named CrRV-Ch01 (accession no: KY203909), was obtained by Sanger sequencing of RNA from *C. rogercresseyi*, using the assembled genome sequence as the basis for primer design. The lice were collected from the skin of farmed Atlantic salmon at three farming sites in Los Lagos (Region X) on the coast of Chile in 2016. The three farming sites are located with an average distance of 65 km. The parasites were fixed in RNAlater® (Thermo Fisher) and transported to the University of Bergen, Norway. RNA was extracted from the lice using TRI Reagent (Sigma-Aldrich), with the following modifications of the standard protocol: The lice were homogenized in 1 ml TRI Reagent with a 5-mm bead for 7 min at 50 Hz in a Tissuelyser LT (QIAGEN), and the RNA pellet was washed with an additional 1 ml of 100% ethanol before air-drying.

RNA from 4-5 pooled lice from one location was ligated to allow circularization and sequencing of the genome termini of CrRV-Ch01. To increase the efficiency of RNA ligation, the 5’-triphosphate residues of the RNA were removed by incubating 5 μg of total RNA with 5 units of 5’ RNA pyrophosphohydrolase (Rpph; New England Biolabs) in 40 μl of 1X NEBuffer 2 for 30 min at 37 °C [18], followed by RNA clean-up using an RNeasy Mini Kit (QIAGEN). Four hundred ng of purified dephosphorylated RNA was then ligated for 1 h at 37 °C using 10 U of T4 RNA ligase (Thermo Scientific) in 20-μl of 1x reaction buffer for T4 RNA ligase supplemented with 0.1 mg of BSA per ml and 40 units of RNaseOUT™ (Invitrogen). For cDNA synthesis, 2.5 μl of ligated RNA was used directly as template for SuperScript™ III reverse transcriptase (SuperScript™ III First-Strand Synthesis System for RT-PCR, Invitrogen), with gene-specific primers annealing to the L gene in the genomic RNA. The cDNA was subjected to nested PCR with forward primers located within the 3’ end of the L gene and reverse primers located within the 5’ end of the N gene (Table 1), using the Expand™ High Fidelity PCR System (Roche). Finally, the nested PCR products were gel purified (QIAquick® Gel Extraction Kit, QIAGEN) and sequenced by the Sanger method.

**Table 1:** Primers and probes for TaqMan real-time RT PCR assays targeting the N protein gene of CrRv-Ch01,18S from *C. rogercresseyi*, and primers used for sequencing of the genome termini

Code	Sequence
CrRV-1-NF	5′-TGG ATT CTT TCC CCC ATT G-3′
CrRV-1-Nprobe	5′-ATC GGA CGA ACT GAT-3′
CrRV-1-NR	3′-TCT TGT CCT GGC TCC ATA GTG-5′
Crog-18sF	5′-AGC GTC TAG CTA CAC GAG ATT GAG-3′
Crog-18sprobe	5′-AAT AAC AGG TCT GTG ATG CC-3′
Crog-18sR	3′-CGT GCA GCC CAG AAC ATC TA-5′
CRRV1_end_fw	5′-TGGAATCTCTCGGGCAAGG-3′
CRRV1_end_fw_nested	5′-TGGATTCGAGTCCTGTCTCG-3′
CRRV1_end_rev	5′-CATCTCGAGTCAATACCTCC-3′
CRRV1_end_rev_nested	5′-CGAAAGCTAACACATCAGGC-3′

The resulting complete virus genome sequence of CrRV-Ch01 differed by 1.1% from the assembled sequence in the GenBank database and contained six open reading frames (ORFs) that were at least 408 nucleotides (136 amino acids) in length, in the order 3’-N-P-M-U-G-L-5’, i.e., different from the standard five-gene arrangement found in many of the rhabdoviruses. The leader region of CrRV-Ch01 is 78 nucleotides long, while the trailer region is 67 nucleotides long. The first 21 nucleotides of the leader region and the last 21 nucleotides of the trailer region exhibit an inverse complementarity of 81.8%. The putative transcription termination/polyadenylation signal (TTP), based on its homology to other rhabdoviruses, is conserved in the genome of CrRV-Ch01 and comprises the motif TATG(A)7. The transcription initiation (TI) sequence, AACAA, is present for all genes except for the U protein gene, which has AATAA as its TI sequence.

The putative nucleoprotein gene (N gene) in CrRV-Ch01 is 1425 nt long and contains a single ORF of 1356 nt encoding a putative protein of 452 amino acids (accession number: APF32073). The putative N protein of CrRV-Ch01 shows 98.3% nucleotide sequence identity (98.0% amino acid sequence similarity) to a partial putative rhabdovirus nucleoprotein sequence (accession no: FK898446, 953 nucleotides) obtained from *C. rogercresseyi* in the Pacific Ocean (Chile). Further amino acid sequence comparisons with other rhabdoviruses using BLAST search showed that CrRV-Ch01 shares 27.7% identity and 48.0% similarity with the virus Lepeophtheirus salmonis rhabdovirus No127 (LSRV-No127) present in *L. salmonis* in the North Atlantic, while the amino acid sequence similarity to both Bivens Arm virus and Tibrogargan virus (genus *Tibrovirus*) is 40.0%. The N protein contains the sequence _283_QLSTRSPYST_292_, which shares high similarity with the RNA-binding motif (G(L/I)SXKSPYSS) present in vesiculoviruses, ephemeroviruses and lyssaviruses [19].

The putative phosphoprotein gene (P gene) of CrRV-Ch01 is 807 nt long and contains a single ORF of 759 nt encoding a putative protein of 253 aa (accession number: APF32074). It contains 35 potential serine/threonine phosphorylation sites and five tyrosine phosphorylation sites. This putative P protein sequence shares no significant sequence identity to any sequences in the GenBank database and its identity is only suggested based on its size and genome position.

The putative matrix protein gene (M gene) in CrRV-Ch01 is 739 nt long and contains a single ORF of 687 nt encoding a putative protein of 229 aa (accession number: APF32075). Amino acid sequence comparison with other rhabdoviruses showed that it shares 24.6% and 25.1% identity and 42.4% and 40.2% similarity with the matrix protein genes of the salmon louse rhabdoviruses LSRV-No127 (AIY25914) and Lepeophtheirus salmonis rhabdovirus No9 (LSRV-No9) (AIY25909), respectively, but no significant match with other rhabdoviruses. A potential domain, PPPY/P(S/T)AP, which is necessary for efficient budding of the virus [20], could be represented by the sequence _14_PVASAPTVPGP_29_.

The putative unknown protein gene (U gene) in CrRV-Ch01 is 439 nt long with a single ORF of 408 nt encoding a putative protein of 136 aa (accession number: APF32076). The U gene nucleotide sequence and the putative protein sequence share no detectable sequence similarity with any other viruses in the public databases. However, topology analysis of the putative U protein using the Phobius server predict an N-terminal signal peptide from aa 1 to 19 and an ectodomain from aa 20 to 136, but no transmembrane region. Additional protein coding genes have been described previously in several rhabdovirus genomes, including genes inserted between the M and G genes [21–29]. They may occur as alternative or overlapping ORFs within the structural genes or as independent ORFs flanked by transcription initiation and termination sequences, as is the case with the putative U protein gene in CrRV-Ch01 [4, 5, 23, 24]. A protein with a signal peptide at this position has been described in tupaia rhabdovirus, TUPV [24]. However, unlike the U protein in CrRV-Ch01 the additional protein in TUPV contain a transmembrane region. A putative protein with a signal peptide and no transmembrane region has been found in Oak Vale virus in the same genomic position as the U protein gene of CrRV-Ch01 [26]. While the function is unknown, the signal peptide suggests that it may be secreted from infected cells.

The glycoprotein gene (G gene) in CrRV-Ch01 is 1647 nt long and contains a single ORF of 1611 nt encoding a putative protein of 537 amino acids (accession number: APF32077). Topology analysis using the Phobius server predicted an N-terminal signal peptide (aa 1–17), an ectodomain from aa 18 to 493, transmembrane region spanning from aa 494 to 516, and a C-terminal cytoplasmic tail from aa 517 to 537. The protein is predicted to contain five putative N-glycosylation sites, _140_NDSD, _242_NKTS, _368_NKSS, _420_NDSS, and _476_NGTS, respectively. BLAST searches showed that CrRV-Ch01 shares the highest amino acid sequence identity (22.3%) with a virus detected in a salmon louse (*L. salmonis*), LSRV-No127 (accession no. KJ958536, unclassified rhabdovirus). The amino acid sequence identity to the classified anguillid perhabdovirus (accession no. AIY29111) is 20.7%.

The L gene of CrRV-Ch01 is 6429 nt long and contains a single ORF of 6390 nt encoding a putative protein of 2130 aa (accession no. APF32078). The L protein gene shows a clear affinity to those of other members of the family *Rhabdoviridae*, with the closest relationships to the L proteins of LSRV-No9 (44.2% identity) and LSRV-No127 (44.0% identity). The vesiculoviruses Maraba virus, Cocal virus, Perinet virus, and Indiana virus are slightly more distant, showing 38.3, 38.3, 38.6, and 38.5% identity, respectively. Six blocks of conserved sequences are shared among the L proteins of members of the family *Rhabdoviridae* [30]. These blocks were also identified in the CrRv-Ch01 L protein after pairwise alignments with L proteins from related rhabdoviruses (data not shown).

To investigate the relationship of CrRV-Ch01 to other members of the family Rhabdoviridae, a phylogenetic tree was generated based on the L protein (Fig. 1). One hundred twenty-five sequences representing all of the genera of the family *Rhabdoviridae* were aligned using MAFFT. Ambiguously aligned regions in the alignment were removed using TrimAl [31], resulting in a sequence alignment of 1571 amino acids. Phylogenetic relationships were determined using RAxML (Randomized Axelerated Maximum Likelihood) v8.2.10 [32], available at CIPRES, employing the LG model of amino acid substitution [33]. The tree was based on approximate maximum-likelihood values using the selected model of substitution and rate heterogeneity. The robustness of each node was determined using 1000 bootstrap steps. The phylogenetic analysis showed that CrRV-Ch01 groups in a clade with two other rhabdoviruses from a parasitic copepod (*L. salmonis*) present on salmon in the North Atlantic. The phylogeny of these rhabdoviruses from copepods shows no clear affinity to members of any of the known rhabdovirus genera.

**Fig. 1:**
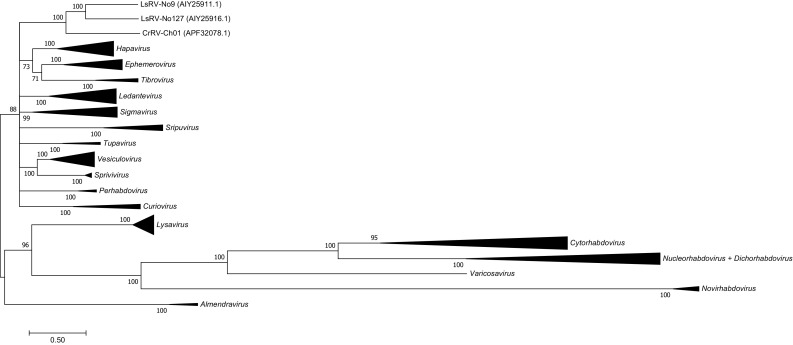
Phylogenetic position of CrRV-Ch01 (accession number: APF32078.1) obtained from *Caligus rogercresseyi* in relation to other rhabdoviruses based on analysis of the L protein sequences after removal of ambiguously aligned regions using TrimAl [31]. The evolutionary relationship is presented as a maximum-likelihood tree based on 1571 aa from the complete alignment of the L protein amino acid sequences with a bootstrap support cutoff of 70%. Branch lengths represent relative phylogenetic distances according to maximum-likelihood estimates based on the LG model of amino acid substitution [33]. The scale bar shows the number of amino acid substitutions as a proportion of the branch lengths. CrRV-Ch01 constitutes a clade together with two rhabdoviruses, LsRV-No9 (accession no: AIY25911.1) and LsRV-No127 (accession no: AIY25916.1), from *L. salmonis*

To estimate the prevalence of CrRV-Ch01 in adult *C. rogercresseyi* from the three locations in Chile, 51 lice were individually tested for the presence of the virus genome sequence, using a qPCR assay targeting the N gene. Two real-time RT PCR assays with TaqMan probes were developed based on the putative nucleoprotein gene sequence obtained from the rhabdovirus CrRV-Ch01, present in *C. rogercresseyi*, and the 18S (small subunit rRNA) from *C. rogercresseyi* (Table 1). Both assays were optimized for real-time RT PCR, and the assay targeting the 18S from *C. rogercresseyi* (Crog-18S) was used as internal control. The real-time RT-PCR reaction was run in a 12.5-μl reaction mixture containing 6.25 μl of 2X RT-PCR, 1.0 μl of 10 mM forward primer, 1.0 of μl 10 mM reverse primer, 0.22 μl of 10 mM probe, 0.25 μl of 25X RT-PCR enzyme mix, 1.75 μl of RNase-free water and 2.0 μl of template. The real-time RT-PCR analysis was run using an Applied Biosystems 7500 Fast Real-Time PCR System under the following conditions: reverse transcription at 45°C for 10 min, polymerase activation at 95°C for 10 min, and 45 cycles of DNA dissociation at 95°C for 15 and annealing/elongation at 60°C for 45 s. The virus was present in both male and female lice from all three locations in Chile. The prevalence of positive lice was 72 % (36 of 50 lice) and ranged from 64.2 to 85.7% for the three locations. The CT value of positive lice ranged from 12.6 to 35.8 and the average CT value was 27.7, ranging from 25.2 to 29.9 for the three locations. Four individuals tested strongly positive (Ct < 15) for the virus. The internal control assay, Crog-18S, gave Ct values ranging from 4.3 to 10.0 (average, 5.7) showing the presence of RNA in all samples. Viral RNA was also detected in egg strings from three positive female lice (Ct values ranging from 30.6 to 31.6). The presence of viral RNA in egg strings could indicate, as described for the *L. salmonis* rhabdoviruses [15, 34], that vertical transmission may occur in addition to horizontal transmission.

To summarize, we have characterized the first rhabdovirus genome from the parasitic copepod *Caligus rogercresseyi,* CrRV-Ch01, collected from farmed Atlantic salmon in Chile. CrRV-Ch01 was not present in all the specimens of *C. rogercresseyi* tested, and we have found what appears to be a complete, functional genome. The completeness of the genome sequence, the presence of complete genome termini exhibiting inverse complementarity, TI and TTP sequences for all genes, and ORFs for all essential rhabdovirus proteins, makes it highly unlikely that this virus is integrated into its host genome. To yield the results of this study, an endogenous virus genome would have to be transcribed as a complete negative-sense viral genome with no post-transcriptional modifications. Thus, this study strongly suggests that CrRV-Ch01 is indeed an exogenous virus rather than another example of endogenous viral elements [8]. To support this, future studies should employ histology, *in situ* hybridization, and electron microscopy to examine the pathology and tropism of the virus and presence of viral particles. The presence of CrRV-Ch01 in Atlantic salmon has not been studied. Because the lice feed on the mucus, skin and blood of their host, it is possible that we have detected a salmonid virus in the gut of the lice. However, the presence of the CrRV-Ch01 genome in the egg strings of the lice strongly suggests otherwise. Nevertheless, future studies should investigate the presence of CrRV-Ch01 in Atlantic salmon and other fish frequently infested with *C. rogercresseyi*. This could reveal if this is an arbovirus infecting both fish and lice, or solely a caligid virus. The closely related LsRV-No9 and LsRV-No127 have been detected in the skin of Atlantic salmon at the attachment site of chalimi, but there are no evidence of infection or replication [15].

The phylogenetic clustering of CrRV-Ch01 from Chile with the rhabdoviruses LsRV-No9 and LsRV-No127 from salmon lice in the North Atlantic, i.e., the demonstration of the presence of these related viruses over a large geographical area, suggest a *Caligidae*-specific association of this virus clade that has probably existed for a considerable time. This indicates that there may be several undiscovered rhabdoviruses infecting other members of the family *Caligidae*.

## Electronic supplementary material

Below is the link to the electronic supplementary material.

Supplementary material 1 (PDF 30 kb)
