# Supplementary figures and images for: The complete genome sequence of CrRV-Ch01, a new member of the family Rhabdoviridae in the parasitic copepod Caligus rogercresseyi present on farmed Atlantic salmon (Salmo salar) in Chile
Source: Arch Virol. 2018 Feb 14;163(6):1657–61. doi: 10.1007/s00705-018-3768-z (PMC5958147; doi:10.1007/s00705-018-3768-z)

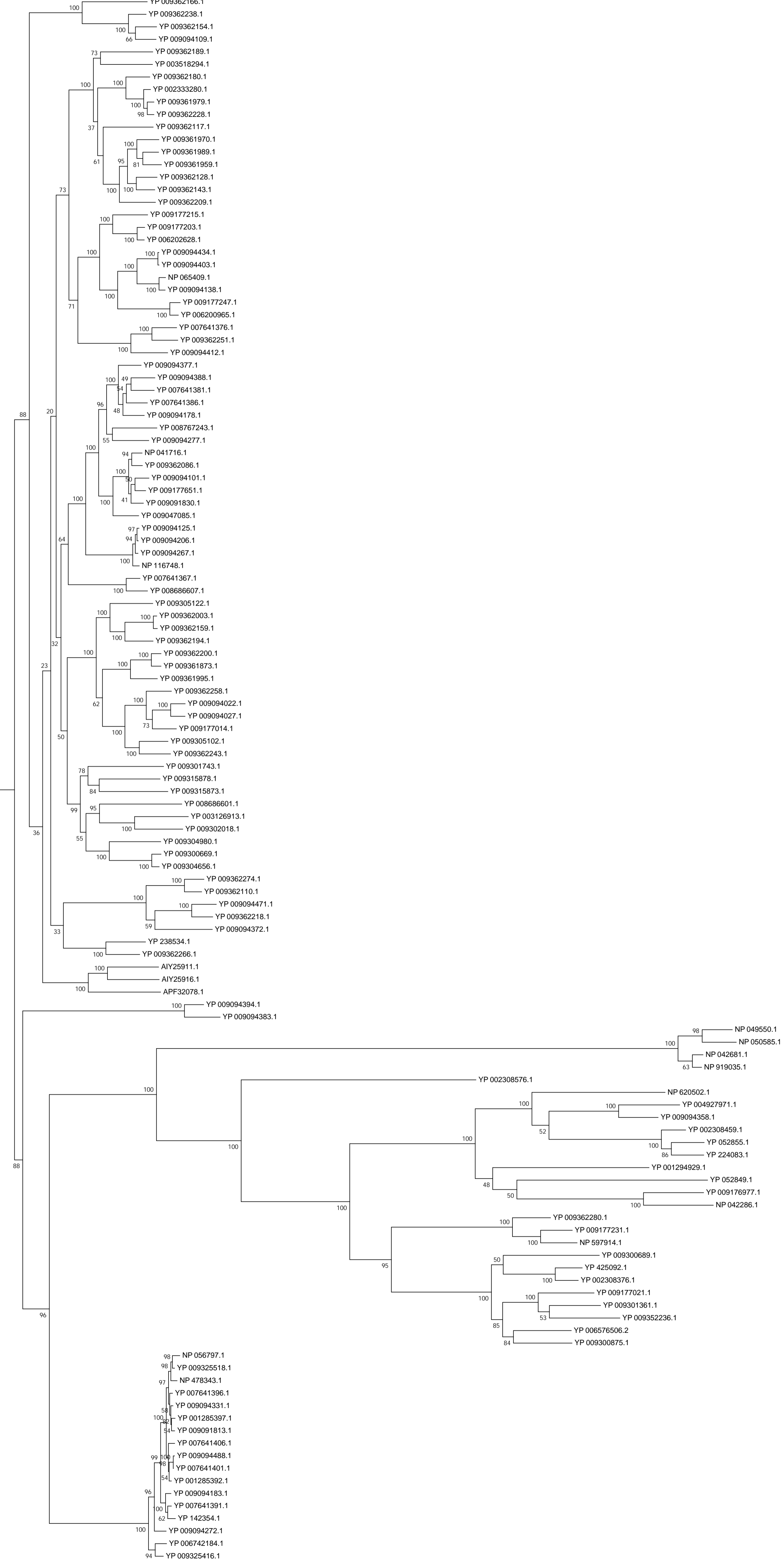

0.50

Supplement: Supplementary file 1 — Supplementary material 1 (PDF 30 kb) [file 705_2018_3768_MOESM1_ESM.pdf]
